# Trends in Loss to Follow-Up among Migrant Workers on Antiretroviral Therapy in a Community Cohort in Lesotho

**DOI:** 10.1371/journal.pone.0013198

**Published:** 2010-10-08

**Authors:** Helen Bygrave, Katharina Kranzer, Katherine Hilderbrand, Jonathan Whittall, Guillaume Jouquet, Eric Goemaere, Nathalie Vlahakis, Laura Triviño, Lipontso Makakole, Nathan Ford

**Affiliations:** 1 Médecins Sans Frontières, Morija, Lesotho; 2 London School of Hygiene and Tropical Medicine, London, United Kingdom; 3 Médecins Sans Frontières, Cape Town, South Africa; 4 Centre for Infectious Disease Epidemiology and Research, University of Cape Town, Cape Town, South Africa; 5 Scott Hospital, Morija, Lesotho; Universidad Peruana Cayetano Heredia, Peru

## Abstract

**Background:**

The provision of antiretroviral therapy (ART) to migrant populations raises particular challenges with respect to ensuring adequate treatment support, adherence, and retention in care. We assessed rates of loss to follow-up for migrant workers compared with non-migrant workers in a routine treatment programme in Morjia, Lesotho.

**Design:**

All adult patients (≥18 years) initiating ART between January 1, 2008, and December 31, 2008, and followed up until the end of 2009, were included in the study. We described rates of loss to follow-up according to migrant status by Kaplan-Meier estimates, and used Poisson regression to model associations between migrant status and loss to follow-up controlling for potential confounders identified a priori.

**Results:**

Our cohort comprised 1185 people, among whom 12% (148) were migrant workers. Among the migrant workers, median age was 36.1 (29.6–45.9) and the majority (55%) were male. We found no statistically significant differences between baseline characteristics and migrant status. Rates of lost to follow up were similar between migrants and non-migrants in the first 3 months but differences increased thereafter. Between 3 and 6 months after initiating antiretroviral therapy, migrants had a 2.78-fold increased rate of defaulting (95%CI 1.15–6.73); between 6 and 12 months the rate was 2.36 times greater (95%CI 1.18–4.73), whereas after 1 year the rate was 6.69 times greater (95%CI 3.18–14.09).

**Conclusions:**

Our study highlights the need for programme implementers to take into account the specific challenges that may influence continuity of antiretroviral treatment and care for migrant populations.

## Introduction

Lesotho (population 1.8 million) is a resource-limited country with a per capita income estimated at $1541 in 2007 [1]. The country's economy is highly dependent on remittances from migrant labourers, mainly temporary mine workers or domestic servants who work in neighbouring South Africa for up to 3 months at a time. Around a third of adult males are estimated to have worked in the mines in South Africa, and migrant workers are thought to account for up to two-thirds of Lesotho's gross domestic product [1], [2].

This high dependency on migrant labour has contributed substantially to the country's HIV epidemic. Lesotho has the third highest HIV prevalence in the world (adult prevalence estimated at 23%) [3], mainly due to vulnerabilities associated with migrant labour such as overcrowded living and working conditions, and long periods away from home leading to disruption of social networks and potential increases in risky behaviours such as alcohol abuse and multiple sexual partners [4], [5].

In 2006, the government of Lesotho embarked on a national programme for the scaling up of treatment and care for people living with HIV/AIDS [6]. Among the many challenges associated with widespread access to HIV/AIDS care in Lesotho, the provision of treatment and care to mobile populations such as migrant workers poses particular challenges. Barriers to care for migrants include access to health services and social support in the host country, discrimination, limited ability to take sick leave, and stigma associated with taking medicine at work [7]. The provision of antiretroviral therapy (ART) in particular raises concerns with respect to ensuring adequate treatment support, adherence, and retention in care. International organisations have highlighted the need to ensure adequate access to prevention, treatment, and care for migrant workers since 2002 [8], but there are few documented outcomes from ART programmes supporting migrant populations.

Médecins Sans Frontières (MSF) has been supporting HIV treatment and care in two districts in Lesotho since 2006. Around 12% of patients in the programme are migrant workers. A rapid survey carried out among a non-random selection of defaulters in 2009 found that the most common reason for defaulting was migration to South Africa for work [3]. We assessed the rates of loss to follow-up for migrant workers compared with non-migrant workers.

## Methods

### Programme setting

MSF and Lesotho's Ministry of Health and Social Welfare established a primary care HIV/AIDS programme in the Scott Catchment Area in 2006. Antiretroviral therapy is provided across 14 primary care health centres and one district hospital. A policy of early initiation of ART was adopted by the government of Lesotho in late 2007 [9]. Patients attend the clinic on a monthly basis for medical check up and ART refills. In exceptional circumstances patients (usually migrant workers) can be given ART supplies for up to 3 months.

### Data collection

Three clinicians extracted data from clinic paper-based registers into an Access database, and this was then exported into STATA (version 11) for analysis. Adult patients (≥18 years) initiating antiretroviral therapy between January 1, 2008, and December 31, 2008, were included in the analysis, and followed up until the end of 2009. Patients were categorised as migrant worker or non-migrant worker according to information collected at treatment initiation: migrants were defined as anyone who crossed to South Africa for the purposes of work (in this context, this included mainly miners, contract workers, and domestic servants). Patients were defined as lost to follow-up if they had not visited a clinic for at least 3 months at the end of the observation period, consistent with common definitions for loss to follow-up in ART programmes. The potential for individuals to return to care (ie, re-enter the cohort) and being counted twice was avoided by taking a thorough treatment history from patients on return, and also the fact that clinics are small (1185 patients across 15 health facilities) and patients are well-known to nurses and lay counsellors.

### Statistical analysis

**Figure 1:**
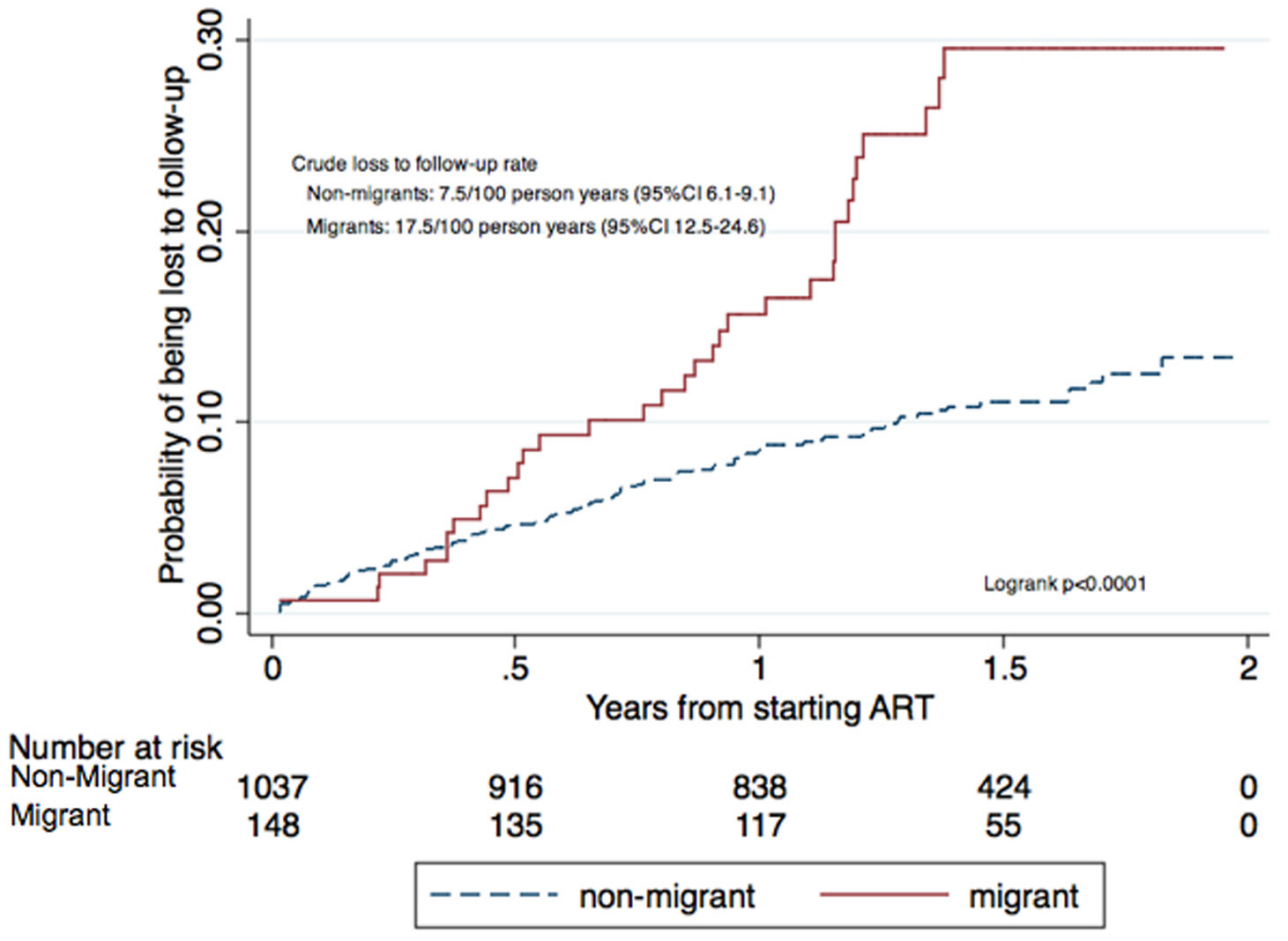
Cumulative hazard estimate for being lost to follow up.

Categorical variables were summarised by percentages and frequencies, and continuous variables by medians and interquartile ranges (IQR). Proportions were compared using the χ^2^ and Fisher's exact test. We described rates of loss to follow-up according to migrant status by Kaplan-Meier estimates (Figure 1), and used Poisson regression to model the association between migrant status and loss to follow-up or death. Endpoints considered for the survival analysis were loss to follow-up, death, transfer, or study end. The Poisson model used Lexis expansion splitting time into 0–3 months, 3–6 months, 6–12 months, and more than 12 months on ART, and was controlled for the following potential confounders identified a priori: age (≤40 or >40 years), sex, baseline CD4 (≤200 cells/mm^3^ or >200 cells/mm^3^), and TB at initiation. All reported p values are exact and 2-tailed, and for each analysis p<0.05 was considered significant.

The analysis was approved by MSF's independent Ethics Review Board [10]. Individual patient consent was not sought because the analysis was based on routine clinical data. All patient information was entered into the database using coded identification numbers, and no information that could reveal patient identity was entered into the database.

## Results

Our cohort comprised 1185 people, of whom 12% (148) were migrant workers. Among the migrant workers, median age was 36.1 (IQR 29.6–45.9) and the majority (55%) were male. Baseline CD4 was slightly lower in migrant workers than in the general population (205 vs 212 cells/mm^3^) but this difference was not statistically significant (p = 0.70) (Table 1).

**Table 1:** Characteristics of migrant and non-migrant patients attending ART services.

		Total patients (n = 1185)	Migrant (n = 148)	Non-migrant (n = 1,037)	p-value
CD4, median (IQR)*		211 (119–284)	205 (94–297)	212 (120–284)	0.70
Sex, n (%)	Female	798 (67%)	66 (45%)	732 (70.6%)	<0.0001
	Male	387 (33%)	82 (55%)	305 (29.4%)	
Age, median (IQR)		39.1 (29.5–48.2)	36.1 (29.6–45.9)	39.2 (31.1–49.7)	0.03
Pregnant at initiation**	Yes	82 (11.6)	3 (4.6%)	79 (10.8%)	<0.0001
	No	653 (88.4%)	63 (95.4%)	653 (89.2%)	
TB at initiation	Yes	198 (16.7%)	198 (16.7%)	168 (16.2%)	0.21
	No	987 (83.3%)	118 (79.7%)	869 (83.8%)	
Baseline Regimen	Tenofovir	607 (51.2%)	100 (67.6%)	507 (48.9%)	<0.0001
	Zidovudine	266 (22.5%)	21 (14.2%)	245 (23.6%)	
	Stavudine	312 (26.3%)	27 (18.2%)	285 (27.5%)	

*data missing for 48 patients.

**denominator includes only women.

After controlling for age, sex, CD4 count at initiation, and TB at initiation, we found that the rates of lost to follow-up were similar for migrant workers and non-migrant workers in the first 3 months, but rates among migrants increased thereafter. Between 3 and 6 months after initiating ART, migrants had a 2.78-fold increased rate of defaulting (95%CI 1.15–6.73); between 6 and 12 months the rate was 2.36 times greater (95%CI 1.18–4.73); whereas after 1 year the rate was 6.69 times greater (95%CI 3.18–14.09) (Table 2).

**Table 2:** Multivariate analysis of rate of loss to follow up at different time points comparing migrants to non-migrants.

		Time on antiretroviral therapy									
		<6 weeks(n = 1137)*		6 weeks–3 months(n = 1089)		3–6 months(n = 1055)		6–12 months(n = 1011)		>12 months(n = 921)	
		Adjusted Rate Ratio (95%CI)	p-value	Adjusted Rate Ratio (95%CI)	p-value	Adjusted Rate Ratio (95%CI)	p-value	Adjusted Rate Ratio (95%CI)	p-value	Adjusted Rate Ratio (95%CI)	p-value
Migrant	No	1		1		1		1		1	
	Yes	0.64 (0.08–4.92)	0.67	1.37 (0.30–6.31)	0.69	2.78 (1.15–6.73)	0.02	2.36 (1.18–4.73)	0.02	6.69 (3.18–14.09)	<0.01
Sex	Female	1		1		1		1		1	
	Male	0.28 (0.06–1.27)	0.90	0.95 (0.84–1.07)	0.36	0.87 (0.78–0.96)	0.01	1.06 (0.56–2.00)	0.85	0.37 (0.15–0.93)	0.04
Age	<40 years	1		1		1		1		1	
	>40 years	0.99 (0.90–1.10)	0.10	0.51 (0.14–1.92)	0.32	0.88 (0.37–2.08)	0.77	0.93 (0.87–0.99)	0.02	0.93 (0.85–1.10)	0.07
CD4 at initiation	≤200 cells/mm^3^	1		1		1		1		1	
	>200 cells/mm^3^	0.29 (0.10–0.85)	0.02	0.46 (0.15–1.44)	0.18	0.75 (0.34–1.68)	0.49	0.72 (0.39–1.34)	0.30	0.81 (0.39–1.71)	0.58
TB at initiation	No	1		1		1		1		1	
	Yes	0.31 (0.04–2.39)	0.26	1.35 (0.36–5.09)	0.65	0.61 (0.18–2.11)	0.44	2.03 (1.03–3.99)	0.04	1.96 (0.81–4.71)	0.13

All variables adjusted for in the final analysis.

*Baseline CD4 counts missing for 48 patients.

## Discussion

Our study found a relatively low rate of defaulting among migrant workers compared to the general population in the first year of treatment, with significant differences observed after one year. Although mobility is recognised as a reason why patients drop out of care [11], we were unable to find other published reports on rates of defaulting among migrant workers on ART for comparison.

Defaulter tracing studies are not conducted routinely in the Lesotho programme, so we are unable to report on the reasons for defaulting. However, one plausible explanation put forward by staff working in the programme is that patients feel less sick and so are able to return to work in South Africa (the “healthy migrant” effect).

There are several limitations to our study. First, outcomes in patients defaulting treatment is unknown. Patients lost to follow up are known to include both those who have sustained an unfavourable outcome (non-access to care, and resulting illness and death) [12], and those whose outcome may be favourable (for example transferring to a more convenient health facility) [13]. The potential for migrant workers to self-transfer to health services in South Africa is unclear. South Africa revised its legislation to allow access to antiretrovirals to foreigners in 2008 [14], but this is reported to be unevenly applied at the health facilities [15]. In areas where migrants are able to access health services in South Africa, outcomes have been highly satisfactory [16]. An additional challenge relates to the lack of harmonisation in treatment guidelines: while Lesotho has recommended a tenofovir-based first-line regimen since late 2007 [17], at the time of our study South African guidelines recommended a stavudine-based first-line treatment regimen (this was revised in 2010). Another limitation, common to all observational studies, is the potential for residual confounding which might have influenced our effect estimate. A further limitation is that we did we did not assess mortality between groups; several deaths will inevitably have occurred among patients lost to follow-up [12], and given the substantial difference in follow-up rates between groups, we considered that any mortality estimate would likely be subject to substantial bias due to misclassification of mortality among patients lost to follow up. We also note that a minority of patients were provided with 3 months' supply of ART. Because our definition of loss to follow-up remained consistent for all patients, a stricter definition was applied to those patients provided with 3-monthly ART (they had only to miss one appointment to be classified as lost to follow-up). We do not consider this would have an important effect since the number of patients provided with 3-monthly ART was small, and this group received additional counselling that stressed the importance of returning to the clinic for ART refills every quarter. Finally, as with all defaulting studies, there is potential that some of the patients considered lost to follow-up will return to care (ie, they will in fact have interrupted their treatment). Although we did not note any instances of treatment interruption, this might be due to the short follow up period: in a recent treatment interruption study done in South Africa the median time of interruption was 228 days [18].

The challenge of ensuring continuity of care for migrant workers has gained increasing attention. In 2009, the Southern African Development Community (SADC) developed a Policy Framework for Population Mobility and Communicable Diseases in the region [19]. The framework identifies a number of challenges for migrant's access health care, including: substantially higher fees for non-nationals; no information as to where services are provided; reluctance among health care providers to provide treatment for communicable diseases requiring long treatment such as TB or AIDS diagnosed in a foreign country; and differing treatment protocols in the different countries [19]. These concerns have been reiterated at the international level: the International Organization for Migration has called for increased measures to ensure access to HIV prevention, treatment, care, and support services are made available for migrant workers by both countries of origin and countries of destination [20].

However, the fact that migrant workers find work in the mines of South Africa poses a significant challenge, since many of the migrant workers are undocumented and find their work in the mines through contractors. The precarious legal status of many of the workers poses an additional barrier to accessing health facilities. Additionally, there are serious gaps in the implementation of TB and HIV programmes in the mines. For example, it has been reported that the gold mining industry has a TB incidence rate up to ten times higher the national incidence rate [21].

SADC has developed a number of policy recommendations to address these challenges of communicable diseases among mobile populations [19]. These include regional harmonisation of drug regimens; coordinated cross border referral services; equitable access to services for mobile populations; coordinated regional public health surveillance and epidemic preparedness; information, education, and health promotion; and operational research on the specific vulnerabilities facing mobile populations. To support these recommendations, the SADC secretariat proposes a number of legal reforms including the protection of foreign workers in high-risk work environments such as mining and agriculture by minimising unfair labour practices and improving work and living conditions. The AIDS Rights Alliance of South Africa (a patient advocacy group) takes these recommendations a step further by calling for the strengthening of existing programmes for the prevention, diagnosis, and treatment of TB and HIV among Basotho mineworkers in South Africa [21]. This would require the private sector to better fulfil its obligations.

Based on the MSF experience of treating mobile populations in conflict-affected settings several practical recommendations can be added. These include: providing drug supplies of up to 3 months for stable patients; adapting adherence counselling to ensure patients are aware of the importance of uninterrupted treatment; developing links with treatment providers in South Africa; and issuing patient-held clinical cards summarising the most important treatment history in case of unplanned self-transfer [22].

In conclusion, our study shows that providing antiretroviral therapy to migrant workers is feasible, and highlights the need for adapted models of care for migrant populations to ensure continuity of antiretroviral therapy. Such support should extend beyond the early phase of antiretroviral treatment initiation which in general populations is considered as the risk period for defaulting from care.
